# Risk factors of prognosis in older patients with severe brain injury after surgical intervention

**DOI:** 10.1186/s40001-023-01473-0

**Published:** 2023-11-04

**Authors:** Hanchao Shen, Haibing Liu, Jiongzhou He, Lianqfeng Wei, Shousen Wang

**Affiliations:** 1 Department of Neurosurgery, The 900th Hospital, Fuzong Clinical Medical College of Fujian Medical University, Fuzhou, 350025 China; 2 Department of Neurosurgery, Cangshan Hospital District of the 900th Hospital, Fuzhou, China

**Keywords:** Risk factors, Older patients, Traumatic brain injury, Surgical treatment, Prognosis

## Abstract

**Background:**

Older patients (aged ≥ 60 years) with severe brain injury have a high mortality and disability rate. The objective of this retrospective study was to assess the clinical risk factors of prognosis in older patients with severe brain injury after surgical intervention and to analyze the prognosis of the surviving group of patients 1 year after discharge.

**Methods:**

Clinical data of older patients with severe brain injury who were admitted to two neurosurgical centers between January 2010 and December 2020 were collected. Patient age, sex, Glasgow Coma Scale (GCS) score at admission, underlying disease, mechanisms of injury, abnormal pupillary reflex, head computed tomography imaging findings (such as hematoma type),intraoperative brain swelling and other factors were reviewed. All the patients were categorized into a good prognosis (survival) group and a poor prognosis (death) group by the Glasgow Outcome Score (GOS); also, the related factors affecting the prognosis were screened and the independent risk factors were identified by the Binary logistic regression analysis. GOS was used to evaluate the prognosis of the surviving group of patients 1 year after discharge.

**Results:**

Out of 269 patients, 171 (63.6%) survived, and 98 (36.4%) died during hospitalization. Univariate analysis showed that age, GCS score at admission, underlying diseases, abnormal pupillary reflex, the disappearance of ambient cistern, the midline structure shift, intraoperative brain swelling, oxygen saturation < 90%, and cerebral hernia were risk factors for the prognosis of older patients with severe brain injury after surgical intervention. Multivariate analysis showed that age, underlying diseases, disappearance of ambient cistern, Oxygen saturation < 90% and intraoperative brain swelling were independent risk factors of the prognosis in the population. The effect of surgical intervention differed among various age groups at 1-year follow-up after surgery.

**Conclusions:**

The results of this retrospective study confirmed that age, underlying diseases, disappearance of ambient cistern, intraoperative brain swelling, and oxygen saturation < 90% are associated with poor prognosis in older postoperative patients with severe brain injury. Surgical intervention may improve prognosis and reduce mortality in older patients (age < 75 years). But for those patients (age ≥ 75 years), the prognosis was poor after surgical intervention.

## Background

Traumatic brain injury (TBI) poses a significant challenge to the healthy life of the older patients. China has more patients with TBI than most other countries [1], and severe TBIs impose a significant economic burden on society and families [2]. Despite the continuous improvement of treatment technology [3–5], the prognosis of older patients (age ≥ 60 years) with severe brain injury remains unsatisfactory, and their mortality and disability rates continue to be high [6, 7].

There is still a lack of unified guidelines for treating older patients with TBI [8, 9]. There are significant differences in treatment management among different centers for older patients with TBI. Some studies show that the prognosis of older patients with severe TBI after surgical intervention is poor [10, 11]. In contrast, other studies emphasize that surgical intervention may positively impact treatment and improve the prognosis of patients [12, 13]. Recent research [6] has found that surgical intervention increased complications and length of hospital stay but was not associated with increased mortality in the older patients with TBI (age ≥ 80 years).

Therefore, this study evaluated the risk factors affecting the prognosis of older patients with severe TBI treated by surgery to understand the impact of surgical intervention on patients’ recovery. This will help make early decisions according to specific information and subsequently make relevant interventions to reduce the mortality of older patients with severe brain injury and improve their quality of life.

## Methods

### Patient population

A total of 281older patients with severe brain injuries admitted to the Neurosurgery Center of the 900th Hospital and the Cangshan Ward of the 900th Hospital between January 2010 and December 2020 were enrolled in this study. The inclusion criteria were: (1) a history of TBI; (2) head computed tomography (CT) examination confirming brain injury; (3) The diagnosis and treatment were in accordance with the guidelines for the Management of Severe Traumatic Brain Injury [14]; (4) Glasgow Coma Scale (GCS) score ≤ 8 points; the patient remained unconscious for more than 6 h or was comatose after waking up (5) completion of surgical treatment; (6) age ≥ 60 years. The following patients were excluded from this study: (1) patients with other intracranial lesions before the injury; (2) patients who abandoned treatment after surgery; and (3) patients with severe heart, lung, and other vital organ failures.

The inpatient records of all patients who met the inclusion criteria were reviewed. These included age, sex, GCS score at admission, mechanisms of injury, cerebral hernia, type of hematoma, underlying diseases, abnormal pupillary reflex, head CT imaging findings (such as subdural hematoma, epidural hematoma, cerebral contusion and/or intracerebral hematoma, and disappearance of ambient cistern), anticoagulant therapy, intraoperative brain swelling, Glasgow Outcome Score (GOS), and other clinical factors.

### Admission management

After admission, two neurosurgeons evaluated all patients. All patients received the same standardized treatment before surgery according to the guidelines for treating TBI. The main surgical techniques were hematoma evacuation and decompressive craniectomy. All patients were treated using standard neurosurgical principles with lowering intracranial pressure and neurotrophic drugs. All patients were treated in the intensive care unit.

### Clinical outcome evaluation

Patients were categorized into survival and death groups by GOS at discharge. GOS was also used to evaluate the outcome of the surviving group 1 year after discharge. Follow-up was conducted via telephone and outpatient visits, and informed consent was obtained from all patients.

### Statistical analysis

All data were analyzed by IBM SPSS Statistics 26. Continuous variables were expressed as medians with interquartile ranges (IQR = Q3–Q1), and categorical variables were expressed as percentages. The Mann–Whitney U test, the chi-square and Fisher’s exact probability tests were used to compare groups. The variables with significant statistical differences (*P* < 0.05) in the single factor analysis were included in the binary logistic regression for multivariate analysis. The conditional independent variables were introduced, and the model was established using the back-off method. For all statistical results, *P* < 0.05 was considered statistically significant.

## Results

### Characteristics of the patient

**Fig. 1:**
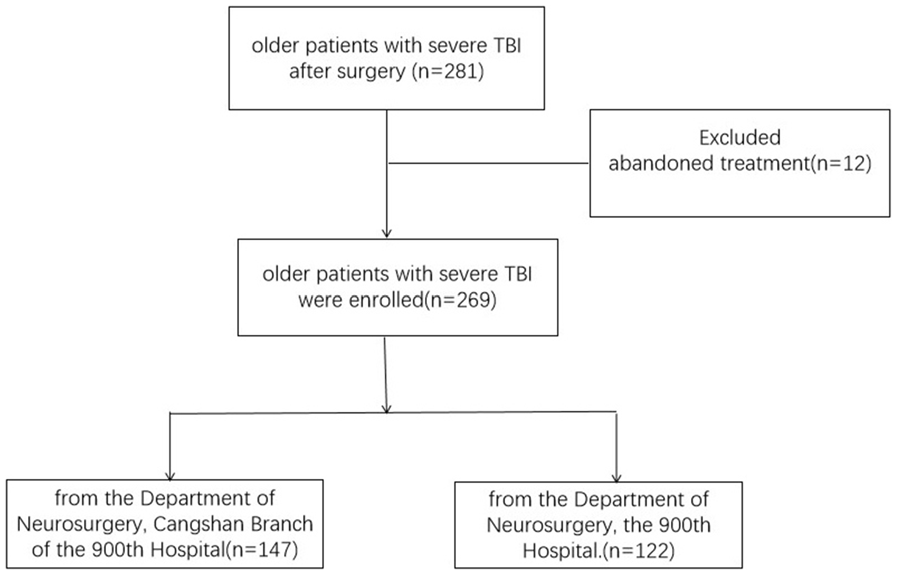
Flow chart of patient selection

A total of 281 patients were enrolled, and 12 were excluded because they abandoned the treatment. The family members gave up treatment during hospitalization and took the patient home because of family economic reasons. Out of 269 patients, 147 were from the Department of Neurosurgery, Cangshan Branch of the 900th Hospital, and 122 were from the Department of Neurosurgery, the 900th Hospital (Fig. 1). There were 209 (77.5%) men and 60 (22.5%) women, and their average age was 72.8 years (Age: from 60 to 89). TBI was caused by traffic accidents in 141 (51.9%) cases, by falls in 111 (41.1%) cases, and by fall from heights in 17 (7.0%) cases. There was no significant difference in the prognosis among the three groups (*P* = 0.086). A total of 193 (71.0%) patients had less than two underlying diseases and 76 (29.0%) patients had more than three underlying diseases, before the injury. The risk of death was higher in older patients with TBI who had three or more underlying diseases, than in those with two or fewer underlying diseases (*P* < 0.001). The clinical characteristics of the patients at admission are shown in Table 1.

**Table 1:** Univariate analysis of prognosis in older patients with severe brain injury after surgery

Factors	Total *n* = 269	Survivor *n* = 171 (63.6%)	Death *n* = 98 (36.4%)	*P* value
Age (years)				
60–74	212 (67.1%)	145 (67.1%)	67 (32.9%)	< 0.001
≥ 75	57 (32.9%)	25 (42.1%)	32 (57.9%)	
Sex				
Male	209 (77.5%)	127 (55.3%)	82 (44.7%)	0.075
Female	60 (22.5%)	44 (71.2%)	16 (28.8%)	
Mechanisms of injury				
Traffic accident	141 (51.9%)	88 (55.8%)	53 (44.2%)	0.086
Fall	111 (41.1%)	76 (66.3%)	35 (33.7%)	
Fall from height	17 (7.0%)	7 (37.5%)	10 (62.5)	
GCS score at admission				
3–5	141 (57.6%)	58 (39.1%)	83 (60.9%)	< 0.001
6–8	128 (42.4%)	113 (85.7%)	15 (14.3%)	
Underlying diseases				
≤ 2	193 (71.0%)	141 (68.3%)	52 (31.7%)	< 0.001
≥ 3	76 (29.0%)	30 (35.8%)	46 (64.2%)	
Types of hematoma				
Subdural hematoma	172 (70.1%)	101 (60.5%)	71 (39.5%)	0.070
Epidural hematoma	15 (5.2%)	12 (75%)	3 (25%)	
Cerebral contusion and Laceration and/or intracerebral hematoma	82 (24.7%)	58 (68.4%)	24 (31.6%)	
Abnormal pupillary response				
Unilateral	168 (58.0%)	145 (83.6%)	23 (16.4%)	< 0.001
Bilateral	101 (42.0%)	26 (24.7%)	75 (75.3%)	
Disappearance of ambient cistern				
No	163 (60.6%)	143 (87.7%)	20 (12.3%)	< 0.001
Yes	106 (39.4%)	28 (26.4%)	78 (73.6%)	
Oxygen saturation < 90%				
No	179 (64.1%)	148 (79.1%)	31 (20.9%)	< 0.001
Yes	90 (35.9%)	23 (22.9%)	67 (77.1%)	
Intraoperative brain swelling				
No	173 (59.7%)	145 (81.9%)	28 (18.1%)	< 0.001
Yes	96 (40.3%)	26 (24.7%)	70 (75.3%)	
The midline structure shift				
≤ 1 cm	154 (52.4%)	136 (86.8%)	18 (13.2%)	< 0.001
> 1 cm	115 (47.6%)	35 (28.2%)	80 (71.8%)	
Using antiplatelet or anticoagulant drugs				
No	165 (61.3%)	112 (67.9%)	53 (32.1%)	0.064
Yes	104 (38.7%)	59 (56.7%)	45 (43.3%)	
Cerebral hernia				
No	54 (20.1%)	49 (90.7%)	10 (9.3%)	0.001
Yes	215 (79.9%)	127 (59.1%)	88 (40.9%)	
Surgical methods				
Hematoma evacuation	123 (45.7%)	87 (70.7%)	36 (29.3%)	0.079
Decompressive craniectomy	44 (16.4%)	26 (59.1%)	18 (40.9%)	
Both	102 (37.9%)	58 (56.9%)	44 (43.1%)	
Median time to surgery in min (IQR)	186 (164–223.5)	184 (164–212)	197 (164–232.3)	0.174
Median duration of surgery in min (IQR)	110 (104–117)	108 (104–116)	112 (104–120)	0.055

### The influence of preoperative use of anticoagulant therapy

Before the injury, 104 (38.7%) patients used anticoagulants such as warfarin, clopidogrel, and aspirin. After surgery, 59 (56.7%) patients survived, and 112 (67.9%) patients survived among the 165 (61.3%) patients who did not receive treatment. There was no significant difference in the prognosis between the two groups (*P* = 0.064). Thus, the use of anticoagulants before injury does not affect disease prognosis.

### The patient’s nervous system was examined after admission

Out of 141 (57.6%) patients with a GCS score of 3–5 points at admission, only 58 (39.1%) patients had a good prognosis, and of the 128 (42.4%) patients with a GCS score of 6–8 points, 113 (85.7%) patients had a good prognosis. The difference was significant (*P* < 0.001). The lower the GCS score at admission, the worse the prognosis. Regarding patients with preoperative unilateral abnormal pupil reactions, 145 out of 168 patients survived after surgery. Conversely, among patients with preoperative bilateral abnormal pupil reactions, only 26 out of 101 patients survived after surgery. The difference were significant between the two groups, and the risk of death was higher in the patients with preoperative bilateral pupil reactions. During emergency treatment, 90 (35.9%) patients with oxygen saturation < 90% at the time of injury underwent endotracheal intubation, and 67 (77.1%) patients in this group died. The difference was significant compared to patients without hypoxia (*P* < 0.001). Patients with an oxygen saturation of < 90% had a higher risk of death.

### Head CT imaging

Preoperative CT revealed 172 (70.1%) cases of subdural hematoma, 15 (5.2%) cases of epidural hematoma, and 82 (24.7%) cases of cerebral contusions and lacerations, intracerebral hematoma, or both. There was no significant difference in prognosis among the three groups (*P* = 0.07). The cisterna ambiens disappeared in 106 (39.4%) patients and survived in 28 (26.4%). The cisterna ambiens did not disappear in 163 (60.6%) patients and survived in 143 (87.7%). The two groups’ prognoses were significantly different (*P* < 0.001). It follows that patients with the disappearance of the cisterna ambiens have a poor prognosis. There were 154 (52.4%) patients with a midline shift ≤ 1 cm, of whom 136 (86.8%) survived, whereas of 115 (47.6%) patients who had a midline shift > 1 cm, only 35 (28.2%) survived. Significant difference was found in prognosis between the two groups (*P* < 0.001). These results suggest that the more obvious the midline structural shift, the worse the prognosis. 127 (59.1%) of the 215 patients with cerebral hernia survived after surgery, whereas of 54 (20.1%) patients without cerebral hernia, 49 (90.7%) survived. The difference in prognosis was significant (*P* = 0.001). It follows that patients with cerebral hernia have a poor prognosis.

### Operative information

Hematoma evacuation was performed in 123 patients and 87 (70.7%) survived, 44 patients underwent decompressive craniectomy and survived in 26 (59.1%). Both procedures were performed on 102 patients and 58 (56.9%) survived. There was no significant difference in prognosis among the three groups (*P* = 0.079). For the Survivor group, the median time to surgery (IQR) was 184 (164–212) mins, the median duration of surgery was 108 (104–116) mins. The median time to surgery (IQR) was 197 (164–232.3) mins and the median duration of surgery was 112 (104–120) mins in the death group. There were no significant difference in these two variables between the Survivor and death group (*P* = 0.174, *P* = 0.055). Brain swelling occurred in 96 (40.3%) patients during the operation, 26 (24.7%) survived, whereas of 173 (59.7%) patients without brain swelling, 145 (81.9%) survived. Between the two groups, the difference in prognosis was significant (*P* < 0.001). Patients with intraoperative brain swelling have poor prognoses.

### Multivariate logistic regression analysis of prognosis in older patients with severe brain injury after surgery

The significant indicators (*P* < 0.05) in univariate analysis such as age, GCS score at admission, underlying diseases, abnormal pupillary response, disappearance of ambient cistern, oxygen saturation < 90%, intraoperative brain swelling, the midline structure shift, cerebral hernia were used for multivariate analysis. The results showed that age, underlying diseases, the disappearance of ambient cistern, oxygen saturation < 90%, and intraoperative brain swelling were significantly associated with poor prognosis (*P* < 0.05) (Table 2). Age, underlying diseases, the disappearance of ambient calcium, oxygen saturation < 90% and intraoperative brain swelling are independent risk factors of the prognosis in older patients with severe TBI after surgical intervention.

**Table 2:** Multivariate Logistic regression analysis of prognosis in older patients with severe brain injury after surgery

Factor	B value	*P* value	Odds ratio	95% Confidence Interval
Age	0.983	0.03	2.673	1.098–6.511
Underlying diseases	1.650	< 0.001	5.209	2.212–12.269
Disappearance of Ambient Cistern	1.548	0.001	4.701	1.949–11.338
Intraoperative brain swelling	2.335	< 0.001	10.332	4.573–23.344
Oxygen saturation < 90%	1.412	0.002	4.106	1.679–10.044

The Hosmer–Lemeshow method was used to test the goodness of fit of the regression model, χ^2^ = 0.641, *P* > 0.05, which could be considered a good fit for the regression model

### Prognosis of older patients with severe brain injury at 1-year follow-up after surgery

Among the 269 patients, 171 patients survived during hospitalization. The 1-year follow-up showed that 102 patients had a good prognosis, and 69 patients had a poor prognosis. Among the age groups, the difference in the prognosis was significant (*P* = 0.005). With the increase in age, the proportion of poor prognosis in each age group gradually increased, and the older the age, the worse the prognosis (Table 3).

**Table 3:** Prognosis of older patients with severe craniocerebral injury of different ages at 1-year follow-up after surgery

Age (years)	Total	Good prognosis (*n*) (GOS 4–5)	Poor prognosis (*n*) (GOS 1–3)	*P* value
60–64	73 (42.7%)	49 (67.1%)	23 (32.9%)	0.007
65–69	43 (25.1%)	28 (65.1%)	17 (34.9%)	
70–74	29 (17.0%)	18 (62.7%)	11 (37.3%)	
75–79	17 (10.0%)	5 (29.4%)	12 (70.6%)	
≥ 80	9 (5.2%)	2 (22.2%)	7 (77.8%)	
	171 (100%)	102 (59.6%)	69 (40.4%)	

GOS: Glasgow Outcome Score

## Discussion

Severe head injury is a high-risk disease in the older population with a long hospital stay and high mortality, which brings great problems to clinical treatment [15–17]. Previous studies had shown that the older the age, the worse the prognosis and the higher the hospital mortality [18–20]. The results of this study show that the poor prognosis of older patients with severe TBI after surgical intervention gradually increased with age especially in patients aged ≥ 75 years, and the proportion of poor prognosis was > 70%. The main reason may be that the older the patient, the more serious the functional degradation and the more postoperative complications and poor prognosis will occur. Age is an independent risk factor for prognosis in older patients with severe craniocerebral injury after surgery. In clinical practice, we should reasonably evaluate the patient’s condition according to age and choose the best treatment to improve the prognosis of patients.

The functions of various organs and immunity in older adults gradually decline with age, and underlying diseases such as hypertension, diabetes, and heart disease also occur. The combination of physiological aging and pathological conditions aggravates the mortality risk of severe TBI in the older population. Compared to younger people, older people have more complications, higher mortality, and longer time for neurological recovery [21, 22]. This study found that, despite active intervention, the mortality of older patients with three or more underlying diseases was much higher than that of patients with two or fewer underlying diseases. The previous underlying disease is an independent risk factor of the prognosis in older patients with severe TBI after surgical intervention and should be considered in clinical practice.

There is no clear conclusion on whether using antiplatelet or anticoagulant drugs before injury affects the prognostic outcome of older patients with severe TBI. Previous studies have found no significant difference in the poor prognosis between TBI patients who used antiplatelet or anticoagulant drug before the injury and those who did not [23, 24]. However, other study shows that Elderly trauma patients using antiplatelet or anticoagulant drugs can lead to elevated risk of intracerebral hemorrhage and poor prognosis [25]. In this study, the use of anticoagulants before injury did not affect the prognosis, and the difference was not significant. However, we found that these patients were more likely to have incomplete intraoperative hemostasis or postoperative hemorrhage, which may increase the difficulty of treating the disease and the length of hospital stay. Therefore, perioperative management is necessary for patients who have received antiplatelet or anticoagulant drugs before the injury.

Because of different degrees of brain atrophy in older patients, the clinical symptoms such as pupil change, slow respiration, and heart rate, and increased blood pressure in the early stage of cerebral hernia are often atypical, which makes it easy to cover up the condition of older patients. For older patients with severe brain injury, when the optimal treatment period is missed, it can cause great difficulties for subsequent treatment. Therefore, early evaluation based on CT images can help predict disease severity as early as possible and facilitate early treatment and intervention. It has been pointed out in the literature [22] that CT images can indicate intracranial lesions in older patients earlier than GCS and provide earlier clinical strategies for clinical treatment. The cistern of the annulus is adjacent to the brainstem. When supratentorial lesions cause acute intracranial hypertension, the morphological changes of the annulus cistern are often the first to appear and it can be used as the early manifestation of cerebral herniation [26]. Patients with the disappearance of the cisterna ambiens after TBI tend to have a deep coma and a poor prognosis. The poor prognosis is mainly because patients with the disappearance of the cisterna ambiens often indicate diffuse intracranial hypertension and secondary brain stem injury, which can lead to deterioration of the patients [27]. Our study showed that the disappearance of cisterns was significantly associated with worse prognosis and higher mortality in older patients with severe TBI after surgical intervention. Therefore, the surgical intervention decision should be made as early as possible according to the width change of the cisterna ambiens. The reduction of the width of the cisterna ambiens represents the possibility of brain stem injury, but the extent to which the width of the cisterna ambiens is reduced as an indication for surgical intervention needs further clinical research.

Older adults are prone to desaturation of blood oxygen because of coma after severe TBI, vomit regurgitation caused by asphyxia, coupled with their cardiopulmonary insufficiency for the decline of their physical function. When older patients experience a TBI, their brain tissue is more sensitive to hypoxia than usual after injury. When the blood oxygen saturation < 90%, the brain cells are severely hypoxic, which aggravates the edema and necrosis and the patient’s condition. This study found that the mortality rate of patients with oxygen saturation < 90% was as high as 87.7%, which was much higher than that of the non-occurrence group. Studies [28] have shown that patients with severe craniocerebral injury have a high proportion of hypoxia during emergency treatment. Therefore, first aid before admission is essential. Suppose the injured older patients with coma can be found in time. In that case, the airway can be opened, and hypoxemia can be corrected before admission. It may reduce the secondary damage caused by brain cell hypoxia, saving time for further treatment. Studies have shown [29] that tracheotomy in the early stages of severe craniocerebral injury can help reduce secondary injury and adverse events in hospitals and increase the chances of early rehabilitation and discharge of patients. Therefore, to improve the airway, increase the oxygen supply of brain tissue cells, and improve the prognosis of older patients with severe brain injury, attention should be paid to airway management in the early stage, and tracheotomy or tracheal intubation should be performed as soon as possible if necessary.

For the older patients with severe TBI, craniotomy hematoma evacuation and decompressive craniectomy are the main surgical procedures [30]. In the process of decompressive craniectomy, there may be complicated by intraoperative brain swelling on the patients with cerebral herniation or high intracranial pressure. Once brain swelling occurs, the prognosis of patients is often poor [31]. This study found a mortality rate of more than 75% in cases with intraoperative brain swelling, significantly higher than in cases without brain swelling (*P* < 0.001). The possible causes of intraoperative brain swelling in older patients with severe brain injury are mainly due to rapid disease progression without early detection and complicated by ischemia–reperfusion injury after decompression [32]. Intraoperative brain swelling in older patients with severe brain injury is a complex pathophysiological process, and further research is needed to confirm the main factors. Thus, reducing the incidence of intraoperative brain swelling in older patients with severe brain injury is important.

Older patients have a poor prognosis with severe TBI. Although active surgical treatment is administered, with an increase in age, postoperative complications and mortality become higher, and their postoperative neurological recovery time also becomes longer. This study showed that patients aged ≥ 75 years had worse postoperative recovery than those aged < 75 years. Therefore, we should perform a reasonable evaluation based on age in clinical practice. Surgical intervention may improve prognosis and reduce mortality in older patients aged < 75 years. For older patients aged ≥ 75 years, surgical intervention may need to be carefully considered.

This study has several limitations. First, this study was retrospective in design. Second, we did not analyze the data on complications. Third, there are few data on the use of antiplatelet or anticoagulant drugs. Therefore, it may be impossible to provide a comprehensive, high-quality data basis for all questions arising from this particular topic. Therefore, further studies are needed to evaluate prognostic factors to provide better and more favorable treatment measures for older patients with severe TBI in the future.

## Conclusions

This study clearly indicates that age, previous underlying diseases, the disappearance of ambient cistern, oxygen saturation < 90% and intraoperative brain swelling are associated with poor prognosis in older patients with severe brain injury undergoing surgical treatment. Surgical intervention may improve prognosis and reduce mortality in older patients aged < 75 years. But for older patients aged ≥ 75 years, the prognosis was poor after surgical intervention. The advantages and disadvantages of surgery should be carefully considered in older patients with severe brain injury of different ages. In clinical practice, a reasonable evaluation should be made according to age. This study further complements previous studies related to surgery in older patients with traumatic brain injury. It provides decision-making and reference for the surgical treatment of older patients with severe TBI, which can help to improve the treatment prognosis and reduce mortality in older patients with severe TBI. The number of older patients with TBI may increase with the aging of the population. Therefore, it is necessary to further study the prognostic factors after surgical treatment for severe brain injury in older adults.

## Data Availability

The dataset supporting the conclusions of this article is included within the article.

## Abbreviations

TBI: Traumatic brain injury
GCS: Glasgow Coma Scale
GOS: Glasgow Outcome Score
ICU: Intensive care unit
CT: Computed tomography
